# Induced pluripotent stem cells derived from the developing striatum as a potential donor source for cell replacement therapy for Huntington disease

**DOI:** 10.1016/j.jcyt.2020.06.001

**Published:** 2021-02

**Authors:** Narawadee Choompoo, Oliver J.M. Bartley, Sophie V. Precious, Ngoc-nga Vinh, Christian Schnell, Ana Garcia, Victoria H. Roberton, Nigel M. Williams, Paul J. Kemp, Claire M. Kelly, Anne E. Rosser

**Affiliations:** 1 Brain Repair Group, School of Biosciences, Cardiff University, Cardiff, UK; 2 Department of Anatomy, Faculty of Medical Science, Naresuan University, Phisanulok, Thailand; 3 Cardiff School of Sport and Health Sciences, Cardiff Metropolitan University, Cardiff, UK; 4 MRC Centre for Neuropsychiatric Genetics and Genomics, School of Medicine, Cardiff University, Cardiff, UK; 5 Wales Brain Repair and Intracranial Neurotherapeutics Unit, School of Medicine, Cardiff University, Cardiff, UK

**Keywords:** cell therapy, epigenetic memory, fetal tissue, Huntington disease, iPSC, striatal differentiation

## Abstract

**Background:**

Cell replacement therapy (CRT) for Huntington disease (HD) requires a source of striatal (STR) progenitors capable of restoring the function lost due to STR degeneration. Authentic STR progenitors can be collected from the fetal putative striatum, or whole ganglionic eminence (WGE), but these tissues remain impractical for widespread clinical application, and alternative donor sources are required. Here we begin exploring the possibility that induced pluripotent stem cells (iPSC) derived from WGE may retain an epigenetic memory of their tissue of origin, which could enhance their ability to differentiate into STR cells.

**Results:**

We generate four iPSC lines from human WGE (hWGE) and establish that they have a capacity similar to human embryonic stem cells with regard to their ability to differentiate toward an STR phenotype, as measured by expression and demethylation of key STR genes, while maintaining an overall different methylome. Finally, we demonstrate that these STR-differentiated hWGE iPSCs share characteristics with hWGE (i.e., authentic STR tissues) both *in vitro* and following transplantation into an HD model. Overall, iPSCs derived from human WGE show promise as a donor source for CRT for HD.

## Introduction

Huntington disease (HD) is a neurodegenerative condition associated with motor, cognitive and psychiatric symptoms [1]. One of the earliest and most prominent neuropathological features is the gradual and progressive loss of striatal (STR) medium spiny neurons (MSNs), with further atrophy occurring in other brain regions as the disease progresses [2,3]. There is currently no cure, and HD patients typically die within 15–30 years of disease onset. Cell replacement therapy (CRT) is a potential medical intervention for HD, aiming to restore function by replenishing the degenerating MSN population. Intra-STR allografts of MSN progenitors derived from primary fetal whole ganglionic eminence (WGE), the primordial striatum, have been shown to express *DARPP-32* (*PPP1R1B*), the principal marker of MSNs, and bring about motor and cognitive functional recovery in HD rat models. Similarly, expression of *DARPP-32* donor-derived cells, along with functional improvements, has been observed in xenografts of human WGE (hWGE) in the HD rat striatum [4]. CRT clinical trials have provided evidence of safety and feasibility of intra-STR transplantation of primary hWGE in HD patients. Furthermore, there is some evidence of long-term graft survival and functional benefits to transplant recipients [5,6]. As such, primary hWGE tissues are currently considered the gold standard donor source for CRT in HD, but they also present a plethora of logistical, quality control and ethical challenges for clinical translation. Hence, there is interest in identifying renewable MSN progenitor sources for clinical application [7].

Human pluripotent stem cells (hPSCs), principally human embryonic stem cells (hESCs) and human induced PSCs (iPSCs), are an appropriate alternative donor source, as they are renewable, accessible for quality control and theoretically capable of differentiating toward an STR fate [4]. There is evidence that iPSCs inherit some epigenetic features associated with the cells from which they were derived, which results in some retained phenotypic features [8,9]. Specific examples include iPSCs derived from insulin-producing β cells [10] and hematopoietic [11], cardiac [12], ocular [13] and neural [14] tissues. Critically, iPSCs derived from forebrain progenitors have been demonstrated to possess an enriched gene expression profile typical of their region of origin, which is reported to enhance their survival following transplantation into the rodent forebrain [15]. Based on the notion that iPSCs can retain an epigenetic profile characteristic of their tissue of origin, we asked whether iPSCs derived from hWGE would be epigenetically primed to differentiate toward STR fates. Specifically, we sought to broadly establish how hWGE-derived iPSCs compared with hWGE in terms of key gene expression markers, epigenetic signatures and engraftment potential.

## Methods

### Ethics and approval

Human fetal tissue was obtained via SWIFT-RTB with full ethical approval and under the UK Human Tissue Authority research license held by Cardiff University (No. 12422). All procedures with animals were performed in full compliance with the UK Animals Act 1986 (Scientific Procedures) and approved by local ethical review.

### Human WGE iPSC generation, culture and validation

Human WGE iPSC generation is described in Supplementary Materials and Methods. In brief, hWGE was dissected and dissociated as previously described [16]. Human WGE iPSCs were generated by transfecting hWGE with piggyBac transposons and transposase (Sanger Institute). Pluripotent-like colonies were identified microscopically, manually picked and then cultured separately as clones, following which integrated transposons were removed [17]. Human PSCs were maintained on irradiated mouse embryonic fibroblasts in ReproStem medium (ReproCell) supplemented with basic fibroblast growth factor (bFGF) (25 µg/500 mL). For spontaneous differentiation, bFGF was removed from culture for 14–21 days. For teratoma formation, hWGE iPSCs (1 × 10^6^) were injected subcutaneously into immunodeficient mice, which were culled at 8 weeks and the tumors dissected.

### STR differentiation

#### Base media

Three types of media were used for differentiation: (i) KnockOut (K/O) medium, as DMEMF/12, KnockOut Serum Replacement (1:100), L-glutamine (200 mM), non-essential amino acid (1:100) and penicillin-streptomycin (1:100) (all Gibco), and β-mercaptoethanol (50 mM; Sigma);

(ii) N2B27 medium, as Advanced DMEM/F12: Neurobasal (1:1), N2 (1:150) and B27 (+RA, 1:150) (all Gibco);

and (iii) N2B27-RA medium, as N2B27 without RA.

#### Protocol

On day 2, hWGE iPSCs were passaged into small clumps and transferred to ReproStem without bFGF to form embryoid bodies (EBs). On day 0, EBs were transferred to K/O medium with SB431542 (10 µM), dorsomorphin (200 µM) (Tocris) and Noggin (500 µg/mL; R&D). On day 5, EBs were transferred to N2B27 medium with *SHH* (100 ng/mL) and *DKK1* (100 ng/mL) (both R&D). On day 10, EBs were plated on Matrigel (Corning) coated plates in N2B27 medium +*SHH*+*DKK1*. On day 16, cells were dissociated with Accutase (Sigma), plated on poly-l-lysine/laminin-coated six-well plates (1 × 10^6^ cells/well) and cultured in N2B27-RA medium +*SHH*+*DKK1*. On day 20, Activin A (25 ng/mL; R&D) was added to N2B27-RA. On day 22, cells were passaged as single cells onto poly-l-lysine/laminin-coated six-well plates (1 × 10^6^ cells/well). From day 30 onward, medium was changed to N2B27 with brain-derived neurotrophic factor (100 ng/mL) and glial cell-derived neurotrophic factor (10 ng/mL) (both PeproTech) as well as ascorbic acid (200 µg/mL) and valproic acid (10 mM) (both Sigma).

### Immunochemistry

Protocol and primary antibodies are described in Supplementary Materials and Methods. Analysis was performed using a Leica DMRBE microscope with Axiocam software.

### DNA methylation analysis

DNA was extracted using a QIAamp DNA mini kit (Qiagen). DNA was bisulfite-converted using an EZ DNA Methylation-Gold kit (Zymo). Bisulfite-converted DNA was processed and quantified using Infinium HumanMethylation450 BeadChips (Illumina) and an iScan system (Illumina). Methylation data analysis was conducted in R software (R Core Team) using the chip analysis methylation pipeline (ChAMP; Bioconductor) [18,19]. All data underwent basic quality control and filtering before analysis as part of the CHAMP pipeline. Data were normalized using the beta-mixture quantile correction [20]. Differentially methylated probe (DMP) analysis was conducted using ChAMP. Multiple comparisons were corrected using the Benjamini-Hochberg false discovery rate correction (0.001). Gene enrichment analysis was conducted using DMP analysis outputs. DMPs were filtered to include only those with a Δβ value difference between groups of ≥0.5. The remaining DMPs were sorted by direction of methylation. Associated genes were compiled using the RefSeq library. These gene lists were analyzed for significant terms in the Allen Brain Atlas upregulated gene library using Enrichr [21,22].

### Polymerase chain reaction and quantitative polymerase chain reaction

Protocol and primer sequences are described in Supplementary Materials and Methods. For statistical analysis, independent *t*-tests were conducted using SPSS 25.0 for each gene analyzed. Data are presented as mean ± standard error of the mean of biological triplicates.

### Electrophysiology

For electrophysiology, recordings and analyses were performed as previously described. Human WGE iPSC STR cells were differentiated using the protocol described above, but K/O and N2B27 media were replaced with the previously described media [23].

### Transplantation

Transplants were conducted as previously described [24]. Briefly, 500,000 cells were transplanted unilaterally into the quinolinic acid-lesioned striatum, and rats received immunosuppression using cyclosporin A (10mg/kg, Sandoz). After 7 weeks, rats were perfused transcardially and the brains collected.

## Results

### Induced PSCs can be generated from hWGE tissues

**Figure 1:**
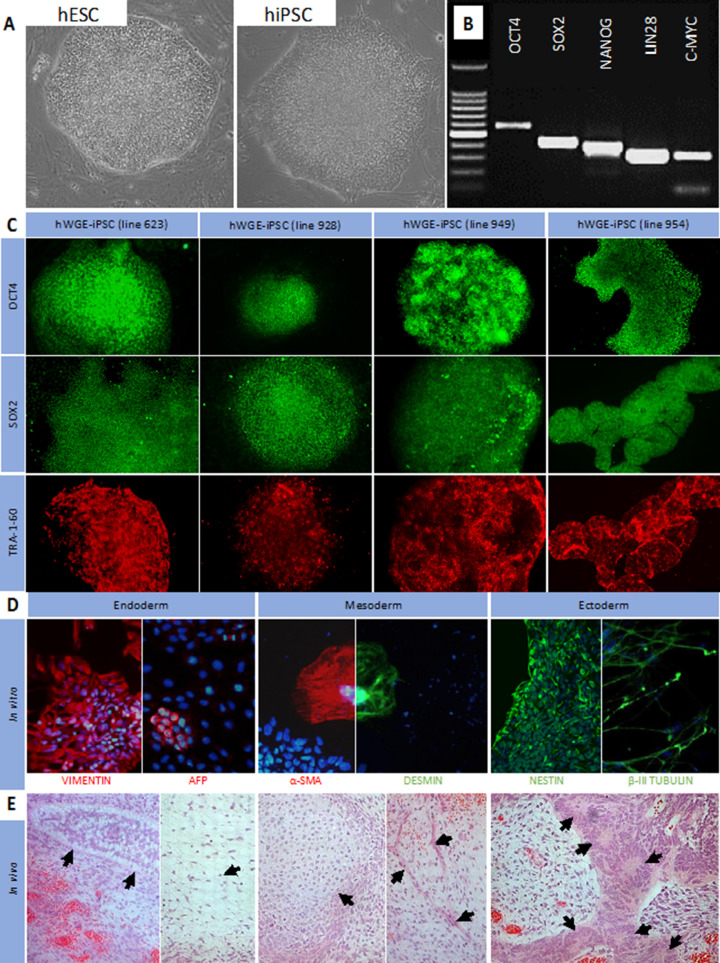
Induced PSCs can be generated from hWGE tissues. (A) Images of hESC and human iPSC colonies. (B) RT-PCR of *OCT4, SOX2, NANOG, LIN28* and *C-MYC* expression in undifferentiated hWGE iPSCs. (C) ICC for *OCT4* (green), *SOX2* (green) and TRA-1-60 (red) in undifferentiated hWGE iPSCs. (D) ICC for endoderm (vimentin, left, red; AFP, right, red), mesoderm (α-SMA, left, red; desmin, right, green) and ectoderm (nestin, left, green; β-III tubulin, right, green) with Hoechst (blue) in *in vitro* spontaneously differentiated hWGE iPSCs. (E) Hematoxylin and eosin staining in teratomas from immune-deficient mice following subcutaneous injection of hWGE iPSCs. Arrows indicate adipose and gland-like tissues (endoderm), chondroblasts and smooth muscle fibers (mesoderm) and neural rosettes (ectoderm). RT-PCR, reverse transcription-polymerase chain reaction; ICC, immunocytochemistry staining; AFP, alpha fetoprotein; SMA, smooth muscle actin. (Color version of figure is available online).

To generate iPSCs, hWGE cells were harvested from four separate human fetal samples (see supplementary Figure 1) and transfected with the piggyBac transposon gene delivery vector system containing the reprogramming genes *OCT4, SOX2, KLF4, C-MYC* and *LIN28*. This resulted in the generation of multiple iPSC colonies from each hWGE sample, and a single clone from each hWGE-derived iPSC population (hWGE iPSC) was then cultured and assessed for pluripotency. All lines exhibited similar cell and colony morphology to hESC controls and were composed of small rounded cells with a high nucleus to cytoplasm ratio (Figure 1A). Pluripotent gene expression was demonstrated across numerous passages in all four lines using RNA analysis (Figure 1B) and immunocytochemistry staining (Figure 1C). Functional pluripotency was demonstrated by derivation of all three germ layers *in vitro* following spontaneous differentiation (Figure 1D) and *in vivo* by teratoma formation in immune-deficient hosts (Figure 1E). Collectively, these data demonstrate successful reprogramming of cells harvested from primary hWGE to a pluripotent state.

### Human WGE iPSCs and hESCs can differentiate toward an STR fate but retain distinct methylation profiles

**Figure 2:**
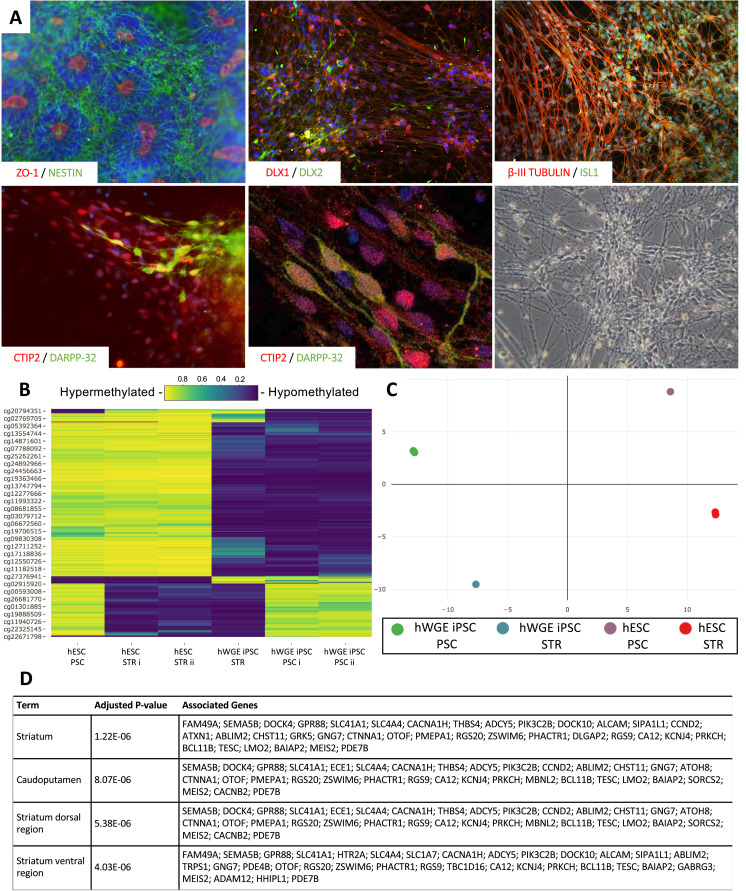
Human WGE iPSCs can differentiate toward an STR fate but retain distinct methylation profiles. (A) Human WGE iPSCs undergoing STR differentiation: neuroectodermal progenitors expressing *ZO-1* (red) and *Nestin* (green) (day 12 to day 14); ventral telencephalon progenitors expressing *DLX1* (red) and *DLX2* (green) (day 14 to day 18); STR progenitors expressing *β-III tubulin* (red) and *ISL1* (green) (day 18 to day 25); MSNs expressing *CTIP2* (red) and *DARPP-32* (green) (day 35 to day 45); and a phase contrast image showing mature neuronal morphology (see also supplementary Figure 2). (B) Heat map and (C) MDS plot of the 1000 most variably methylated probes between undifferentiated PSCs and differentiated STR cells. (D) Results of gene enrichment analysis of differentially methylated genes between PSC (n = 3) and STR (n = 3) samples, showing the top four most significant associated terms in the Allen Brain Atlas upregulated gene library, adjusted *P*values and associated genes. MDS, multidimensional scaling. (Color version of figure is available online).

Using established principles of STR differentiation, we tested the capacity of hWGE iPSCs to differentiate toward an STR phenotype and included hESCs as a control. Successful fate specification was confirmed using immunocytochemistry staining (Figure 2A; also see supplementary Figure 2). In brief, dual SMAD inhibition led to the formation of rosette structures typical of neuroectodermal progenitors, identifiable by their flower-like morphology, and expression of *ZO-1* at the luminal site and *Nestin* (*NES*) in the cytoplasm. Commitment to a neuronal fate was confirmed by expression of *β-III tubulin* (*TUBB3*) and *MAP2*. Patterning toward a ventral telencephalon progenitor fate was initiated using *SHH* and *DKK1* and confirmed by expression of *DLX1* and *DLX2*. Commitment to an MSN progenitor fate was induced through exposure to Activin A. STR progenitor fate commitment was confirmed by expression of *ISL1, GSX2* and *FOXP1*. Following maturation, MSNs were identified by positive co-expression of critical MSN markers *CTIP2* (*BCL11B*) and *DARPP-32*.

We then explored differences in CpG DNA methylation between hWGE iPSCs and hESCs both at a pluripotent stage and following subsequent STR differentiation. Differences in CpG methylation were seen between hWGE iPSCs and hESCs, with more extensive hypomethylation in hWGE iPSCs (Figure 2B). Following STR differentiation, both cell types exhibited demethylation changes in similar regions, although the previously observed methylation differences between PSC sources remained largely unchanged (Figure 2B). When compiled into a multidimensional scaling plot, these differences separated samples into four distinct groups: pluripotent hWGE iPSCs, pluripotent hESCs, STR-differentiated hWGE iPSCs and STR-differentiated hESCs (Figure 2C). Thus, following exposure to an STR differentiation protocol, despite obvious sustained methylation differences, both hWGE iPSCs and hESCs acquired similar demethylation changes. A subset of these changes were revealed by gene enrichment analysis to be significantly associated with terms related to the striatum and its substructures (Figure 2D). Collectively, this demonstrates that hWGE iPSCs respond similarly to key STR differentiation cues previously applied to hESCs and exhibit a similar capacity for STR differentiation, yet there remain epigenetic differences between these cell sources.

### STR-differentiated hWGE iPSCs share characteristics with hWGE

**Figure 3:**
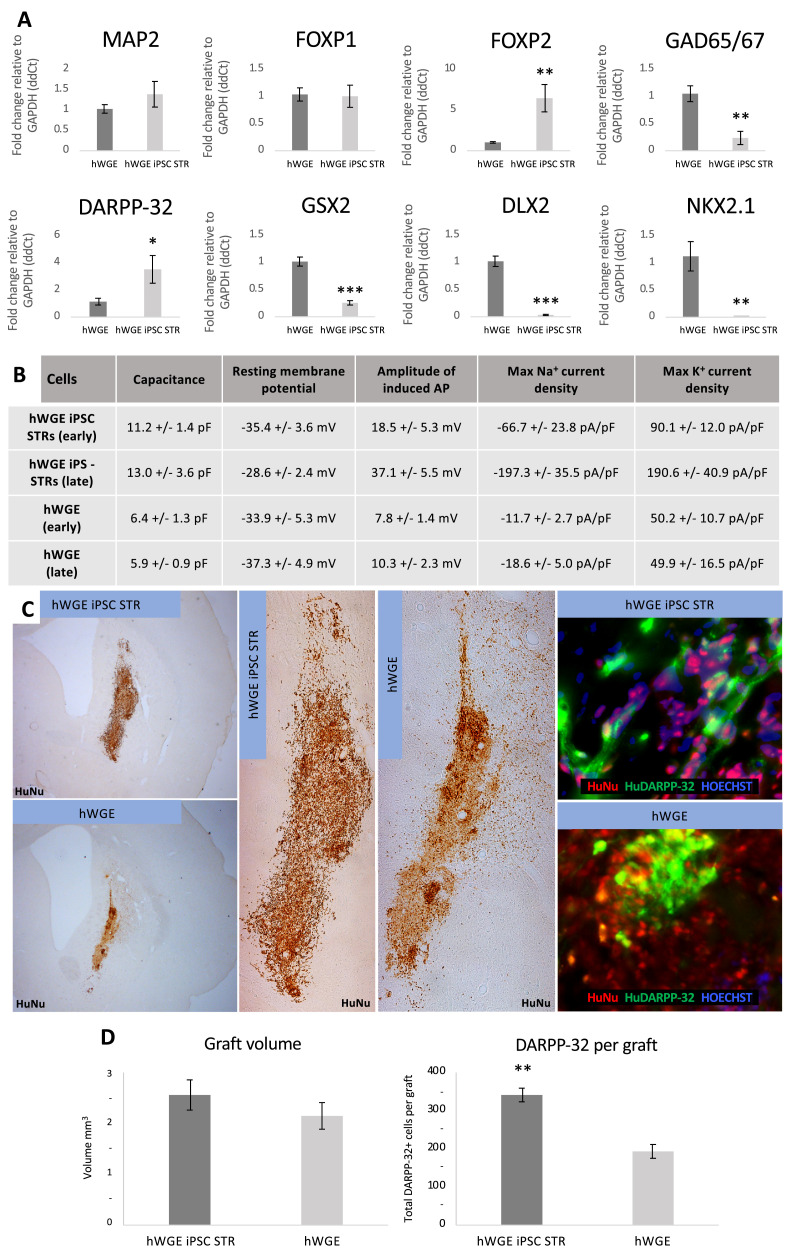
STR-differentiated hWGE iPSCs share characteristics with hWGE. (A) QPCR analysis showing relative gene expression in hWGE iPSC STR cells (n = 5) relative to hWGE (n = 6). (B) Electrophysiological assessment of hWGE iPSC STR cells and hWGE (see also supplementary Table 3). (C) Images of hWGE iPSC STR cells and hWGE grafts: immunohistochemical staining for HuNu (brown) and immunofluorescent staining for HuNu (red) and human-specific *DARPP-32* (huDARPP-32, green). (D) Graft volume and counts of *DARPP-32* cells per graft. *P< 0.05, **P< 0.01, ***P< 0.001. HuNu, human nuclear antigen; QPCR, quantitative polymerase chain reaction. (Color version of figure is available online).

We next sought to compare hWGE iPSCs following STR differentiation (hWGE iPSC STR) with cells derived from hWGE (authentic STR cells). RNA analysis (Figure 3A) revealed similar levels of pan-neuronal marker *MAP2* (t_9_ = –1.173, *P* = 0.105), suggesting similar neuronal composition. Between the two cell types there was no significant difference in *FOXP1* expression (t_9_ = 0.145, *P* = 0.398). However, hWGE iPSC STR cells expressed greater levels of *FOXP2* (t_9_ = –3.567, *P* = 0.006) and *DARPP-32* (t_9_ = –2.473, *P* = 0.035), whereas hWGE expressed greater levels of *GAD65/67* (t_9_ = 4.173, *P* = 0.002). *GSX2, DLX2* and*NKX2-1* are known to be upregulated in the ventral telencephalon, but their expression is reduced in mature MSNs. All three were expressed at significantly lower levels in the hWGE iPSC STR cells (*GSX2* t_9_ = 7.662, *P* < 0.001; *DLX2* t_9_ = 9.307, *P* < 0.001; *NKX2-1* t_9_ = 3.743, *P* = 0.005), implying maturation of these cells.

We also explored potential functional differences between these cell types using patch clamp electrophysiological analysis at early (21–30 days) and late (42–45 days) time points (Figure 3B; also see supplementary Table 3). A higher percentage of hWGE iPSC STR cells displayed induced action potentials (APs) compared with hWGE (hWGE iPSC STR, early 89%, late 100%; hWGE, early 70%, late 40%), and the amplitude of induced APs was higher in hWGE iPSC STR cells than in hWGE. A single spontaneous AP was seen in hWGE iPSC STR cells, but none was seen in hWGE. The output values for the resting membrane potential evaluations (typically in the range of –50 mV to –70 mV in more mature neurons) indicated low levels of maturity in all neurons (hWGE iPSC STR, early –35.4 ± 3.6 mV, late –28.6 ± 2.4 mV; hWGE, early –33.9 ± 5.3 mV, late –37.3 ± 4.9 mV; all values mean ± standard error of the mean). Thus, the electrophysiological analysis suggested overall neuronal immaturity, although this was less marked in the hWGE iPSC STR cells.

Finally, either hWGE iPSC STR cells or hWGE was transplanted into the lesioned rat striatum, and grafts were collected 7 weeks post-transplantation for immunohistochemical analysis. Graft survival and volume were analyzed using human nuclear antigen, a human-specific marker (Figure 3C). No significant differences in volume were found in grafts derived from either hWGE iPSC STR cells or hWGE (*P* = t_15_ = 2.95, *P = 0.086*) (Figure 3D). However, a significantly higher proportion of the grafted hWGE iPSC STR cells were positive for the MSN marker *DARPP-32* (human-specific) (t_15_ = 33.75, *P* < 0.001) (Figure 3D). Subsequently, hWGE iPSC STR-derived grafts appeared to have yielded a greater number of mature MSNs per unit volume than hWGE-derived grafts.

## Discussion

There is a need to find alternative donor cell sources (most likely PSCs) for future application of CRT for HD. It is known that iPSCs retain some epigenetic features of their tissue of origin and that this can enhance differentiation of fates associated with the tissue of origin [8,9]. Thus, iPSCs derived from hWGE could harness their residual epigenome to enhance STR fate differentiation. Here we take the essential first steps to explore this potential donor source.

We have demonstrated the first successful generation of iPSC lines from four separate hWGE tissues and validated their pluripotency. Using developmental principles previously applied to hESCs for STR differentiation [25–[26], [27]], we found that these hWGE iPSCs differentiate into neurons expressing a range of MSN markers, including *FOXP1, CTIP2* and *DARPP-32*, similar to what is achievable with hESCs. We also examined the methylome of hWGE iPSCs and hESCs exposed to the STR differentiation protocol and demonstrated significant demethylation at CpG sites associated with STR genes in both, consistent with the current understanding of the developmental role of methylation [28]. However, although hWGE iPSCs and hESCs appeared to have differentiated into equivalent STR phenotypes in terms of selected STR markers and the demethylation of STR genes, obvious methylome differences persisted. As mentioned, it has been established that iPSCs can retain an epigenetic memory of their tissue of origin, which enhances their capacity to differentiate toward cell fates associated with that original tissue. Human WGE iPSCs may benefit from an epigenetic memory retained from their STR origin, and this epigenetic background may allow such cells to be more suitable for STR differentiations than hESCs. However, elucidating the functional consequences of the retained methylome differences observed here, or even how the observed demethylation of STR genes compares with that of normal development, will need to be accomplished in future work by directly comparing hWGE iPSC lines with human iPSCs derived from non-STR tissues, hESCs and hWGE tissues.

We next compared hWGE iPSC STR cells with an authentic source of MSNs: hWGE. Gene expression analysis of *in vitro* cultures suggested that hWGE iPSC STR cells were overall more mature than their hWGE counterparts. Specifically, despite similar levels of *MAP2* and *FOXP1* expression, we found significantly higher *DARPP-32* expression in the hWGE iPSC STR cells. Conversely, significantly higher expression of earlier ventral telencephalic markers *GSX2, DLX2* and *NKX2-1* were observed in hWGE samples. Electrophysiological analysis corroborated these findings. Human WGE iPSC STR cells exhibited a greater number of APs and at higher amplitudes than the hWGE-derived cells, although in all cases the amplitudes were lower than anticipated in fully mature neurons.

Following intra-STR transplantation into an HD model, we observed significantly greater numbers of *DARPP-32*-expressing cells in hWGE iPSC STR grafts compared with hWGE grafts, despite graft volumes being similar. We cannot comment on the functionality of these grafts, although we can confirm the readiness of hWGE iPSCs to differentiate into MSNs *in vivo*. There are a number of potential explanations as to why these grafts appear to be enriched for *DARPP-32*-positive cells compared with hWGE. For example, STR differentiation is designed to produce MSN progenitors, rather than a full complement of hWGE cells (e.g., STR interneurons), which is consistent with the higher expression of *GAD65/67* (expressed by both MSNs and STR interneurons) observed in hWGE. Alternatively, these differences could result from the variances in maturity suggested by our *in vitro* data, and further *in vivo* graft maturation could perhaps alleviate these differences.

In summary, although hWGE iPSCs appear to possess an STR differentiation capacity similar to that of hESCs, their methylation profiles remain distinct. It is possible that these differences represent an epigenetic memory of tissue of origin, which could enable hWGE iPSCs to produce MSNs that are more similar to their original tissue: hWGE. We have established that hWGE iPSCs produce MSNs that share fundamental characteristics with hWGE-derived MSNs, thus confirming their potential as a useful alternative cell source for CRT for HD and offering evidence that such cell lines may be capable of overcoming the limitations of fetal tissues.

## Declaration of Competing Interest

The authors have no commercial, proprietary or financial interest in the products or companies described in this article.

## Appendix Supplementary materials

Supplementary: Supplementary materials and methods. Supplementary Figure 1. hWGE dissection and hiPSC generation information. Supplementary Figure 2. Additional ICC from in vitro STR differentiation. Supplementary Table 3. Electrophysiological analysis.
