# Supplementary material for: Induced pluripotent stem cells derived from the developing striatum as a potential donor source for cell replacement therapy for Huntington disease
Source: Cytotherapy. 2021 Feb;23(2):111–8. doi: 10.1016/j.jcyt.2020.06.001 (PMC7822401; doi:10.1016/j.jcyt.2020.06.001)
Supplement: Supplementary file 1 [file mmc1.docx]

# Supplementary Materials and Methods

## hWGE-iPSC generation & validation

**Day -7**, Human fetal WGEs were dissected, dissociated (Roberton et al., 2017) and seeded on PLL/laminin coated 6-well plates (5x10^5^ cells per well) in plating medium (DMEMF/12, FCS). **Day 1**, cells were transfected with piggybac transposons and transposase (0.5µg of pPB-CAG.OSKML-puΔtk and 0.5µg of pCyL43 (Sanger Institute)) using Lipofectamine 2000 (Invitrogen). **Day 2**, cells were dislodged and dissociated using Accutase (Sigma) and re-plated on irradiated mouse embryonic fibroblasts (MEFs) in plating medium. **Day 3**, medium changed to ReproStem (ReproCell). Colonies appeared and were picked between **Day 5-14**. Transposon integration was removed prior to further expansion (Yusa et al., 2009). For spontaneous differentiation, hWGE-iPSCs were plated on matrigel coated coverslips and cultured in ReproStem without bFGF for 14-21 days. For teratoma formation, hWGE-iPSCs (1x10^6^ in 50 μl ReproStem) were mixed 1:1 with 50 μl Matrigel (corning) and injected s.c. into immunodeficient mice. 8 weeks later, tumors were dissected and processed.

## Culture of PSCs

All hPSCs were maintained on MEFs in ReproStem supplemented with bFGF (25µg/500ml). All lines were passaged manually by incubating with collagenase IV (Gibco) and using a flame pulled glass pipette to cut and harvest the colony. hESC line H9 was purchased from (Wicell).

## Culture of hWGE

hWGE was cultured on PDL treated plates in *NDM medium*: DMEMF/12, FCS (1:100), B27 (+RA) (1:100), and Pen/Strep (1:100) (all Gibco).

## Immunochemistry

Cells and brain tissue were first blocked in 3% normal serum for 1 hour, then incubated in primary antibody with 3% serum overnight. The next day, cells and tissue were incubated in secondary antibody (1:200) with 3% serum for 2 hours. For fluorescent stains, Alexa Fluor secondary antibodies (Life Technologies) and a Hoechst stain was used. For DAB stains, sections were incubated in an avidin-biotinylated enzyme complex (Vector Laboratories, Peterborough, UK) for 2 hours, then a DAB colour reaction was used.

The primary antibodies used in this study were: OCT4 (1:500; Abcam, ab19857), SOX2 (1:500; Abcam, ab97959), TRA-1-60 (1:200; Abcam, ab16288), VIMENTIN (1:200; Millipore, MAB3400), αSMA (1:200; Dako, M0851), FOXP1 (1:200; Abcam, ab32010), CTIP2 (1:200; Abcam, ab70453), ZO-1 (1:200; BD Bioscience, BD610966), β-III TUBULIN (1:1000; Sigma, T2200), NESTIN (1:500, Millipore, ABD96), HuNu (1:1000; Millipore, MAB1281), DARPP-32 (1:500; Abcam, ab40802).

## PCR & QPCR

Total RNA was extracted using RNeasy Mini-Prep (Qiagen). Reverse transcription was performed using SuperScript IV First-Strand Synthesis System (Invitrogen). PCR was conducted using BioTaq DNA polymerase (Bioline). QPCR was conducted using PowerUp^TM^ SYBR^TM^ Green (Invitrogen). StepOnePlus^TM^ Real-Time PCR System (Applied Biosystems) was used for PCR and QPCR. All QPCR data are analysed relative to two reference genes: GAPDH and β-ACTIN. Data are presented as mean + s.e.m. of biological triplicates.

**Primer sequences:**

OCT4: GACAGGGGGAGGGGAGGAGCTAGG, CTTCCCTCCAACCAGTTGCCCCAAAC;

SOX2: GGGAAATGGGAGGGGTGCAAAAGAGG, TTGCGTGAGTGTGGATGGGATTGGTG;

NANOG: CAGCCCCGATTCTTCCACCAGTCCC, CGGAAGATTCCCAGTCGGGTTCACC;

LIN28: GAAGCGCAGATCAAAAGGAG, TGCACCCTATTCCCACTTTC;

C-MYC: GCGTCCTGGGAAGGGAGATCCGGAGC, TTGAGGGGCATCGTCGCGGGAGGCTG.

β-ACTIN: TCGTGCGTGACATTAAGGAG, GTCAGGCAGCTCGTAGCTCT;

GAPDH: CCTCAACGACCACTTTGTCA, TTACTCCTTGGAGGCCATGT;

MAP2: AAAGCTGATGAGGGCAAGAA, GGCCCCTGAATAAATTCCAT;

GAD67: CCAAGGTGCTGGACTTTCAT, AAATCGAGGATGACCTGTGC;

FOXP1: CGATCCCTTCTCTGATTTGC, CATGCATAATGCCACAGGAC;

FOXP2: CCACGAAGACCTCAATGGTT, GCTCTTCCTTGACGTGGATT;

DARPP-32: CTGGGCAAAAGACAACCTGT, GGTCTTCCACTTGGTCCTCA;

DLX2: AGCAGCTATGACCTGGGCTA, TCCTTCTCAGGCTCGTTGTT;

GSX2: AGATTCCACTGCCTCACCAT, CAGGAGTTGCGTGCTAGTGA;

NKX2-1: ATGTCGATGAGTCCAAAGCA, CTCCATGCCCACTTTCTTGT.

## Animal procedures

Transplantation was conducted as previously described (Lelos et al., 2016). In summary, adult female Sprague-Dawley rats (Charles River) were lesioned unilaterally using quinolinic acid (90 mM in PBS) at stereotaxic coordinates (all in mm): from bregma AP -0.4, ML -3.7, below dura DV -5.0 & -4.0; and AP +1.2, ML -2.9, DV -5.0 & -4.0. Six days following lesion surgery, rats began chronic immunosuppression using cyclosporin A (Sandoz Pharmaceutical) at 10mg/kg/day in 0.9% saline, delivered i.p. until the end of the experiment. Seven days following lesion surgery, rats received 250,000 cells at two depths (500,000 cells total), at stereotaxic coordinates AP +0.4, ML -3.2, DV -4.5 & -3.7. After 7 weeks, rats were terminally anesthetized using Euthatal (Merial) and perfused transcardially with 0.01 M PBS followed by 1.5% PFA (Sigma-Aldrich). Brains were postfixed for 24 h in 1.5% PFA, and then sectioned on a freezing sledge microtome at 40 μm thickness in a 1:12 series.

## Supplementary Figure 1. hWGE dissection and hiPSC generation information

(A) Schematic of fetal hWGE dissection, following reflection of cortical flap laterally to reveal WGE (elongated heart shape structure), which is separated from the cortex by undercutting (image adapted from Dunnett and Bjorklund, 1992). Table summarizing hiPSCs derived from 4 separate fetal hWGE samples.

## Supplementary Figure 2. Additional ICC from in vitro STR differentiation

Representative brightfield images and photomicrographs of cells following ICC for specific cell markers. (A) hWGE-iPSCs. Brightfield image of neural precursors. Fluorescent immunocytochemistry for MAP2 (red) and GSX2 (green), with HOECHST (blue). Left is mature, right is immature. (B) hESCs. Brightfield images of neural precursors and mature MSNs. Fluorescent immunocytochemistry for ISL1 (red), DLX1 (green), β-III TUBULIN (red), FOXP1 (green), CTIP2 (red), and DARPP-32 (green), with HOECHST (blue). (C) hWGE. Fluorescent immunocytochemistry for β-III TUBULIN (red), FOXP1 (green), CTIP2 (red), and DARPP-32 (green), with HOECHST (blue).

##

## Supplementary Table 3. Electrophysiological analysis

Complete table of electrophysiological assessment.
